# Does Having Children Affect Adult Smoking Prevalence and Behaviours at Home?

**DOI:** 10.1186/1617-9625-1-16

**Published:** 2003-09-15

**Authors:** AK Johansson, A Halling, The LinQuest Study Group

**Affiliations:** 1 Division of Paediatrics, Department of Molecular and Clinical Medicine, Linköping University, Linköping Sweden; 2 Department of Health Sciences, Kristianstad University, Kristianstad, Sweden; 3 The LinQuest Study Group: eds. Ekberg K, Brage HN, Datserri M, Division of Preventive and Social Medicine and Public Health Science, Department of Health and Society, Linköping University, Linköping, Sweden

## Abstract

**Background:**

Smoking prevalence and smoking behaviours have changed in society and an increased awareness of the importance of protecting children from environmental tobacco smoke (ETS) is reported. The aim of this study was to find out if smoking prevalence and smoking behaviours were influenced by parenthood, and if differences in health-related quality of life differed between smoking and non-smoking parents.

**Methods:**

Questionnaires were sent to a randomly selected sample, including 1735 men and women (20–44 years old), residing in the south-east of Sweden. Participation rate was 78%. Analyses were done to show differences between groups, and variables of importance for being a smoker and an indoor smoker.

**Results:**

Parenthood did not seem to be associated with lower smoking prevalence. Logistic regression models showed that smoking prevalence was significantly associated with education, gender and mental health. Smoking behaviour, as well as attitudes to passive smoking, seemed to be influenced by parenthood. Parents of dependent children (0–19 years old) smoked outdoors significantly more than adults without children (p < 0.01). Logistic regression showed that factors negatively associated with outdoor smoking included having immigrant status, and not having preschool children. Parents of preschool children found it significantly more important to keep the indoor environment smoke free than both parents with schoolchildren (p = 0.02) and adults without children (p < 0.001). Significant differences in self-perceived health-related quality of life indexes (SF-36) were seen between smokers and non-smokers.

**Conclusion:**

As smoking behaviour, but not smoking prevalence, seems to be influenced by parenthood, it is important to consider the effectiveness of commonly used precautions when children's risk for ETS exposure is estimated.

## Introduction

In the European region, smoking, which is influenced by cultural and sociodemographic factors [1], has stabilised during the last decade at approximately 30% for adults [2]. However, the variations between countries are great, and in Sweden the smoking prevalence has decreased to 19%, the lowest in Europe.

The declining smoking trend is even more obvious among pregnant women and among parents of infants [3]. Twelve percent of Swedish pregnant women were smokers in 2000, compared to 25% in 1990 [4]. The same positive trend is reported from the United States, where smoking prevalence among pregnant women has decreased from 16% in 1987 to 12% in 1996 [5]. This is a promising development but not satisfactory, as it has been shown that most mothers resume smoking shortly after giving birth [6].

Interventions are done both on public and individual levels. Common ways developed by society in many countries to strangle tobacco use and minimise ETS pollution in different settings include taxes on tobacco products, restrictions on marketing of tobacco, warning labels on tobacco products, legal restrictions on smoking in public places (schools, theatres, public transport, etc.) and restrictions on buying tobacco [2]. In this respect, however, the home is a more difficult target for tobacco control, although it has been found to be the main source of children's ETS exposure [10]. Different health promotion activities, including tobacco issues, are in use during childhood in Sweden today, starting with antenatal care and proceeding through child health care and school health promotion programs, reaching practically all children aged 0–19 years as well as their parents. During the last decade intensified efforts have been made to develop effective methods to help parents-to-be and parents to stop smoking or change their smoking behaviour [11,12].

The exposure of children to environmental tobacco smoke (ETS) enhances the risk of negative health effects [7] and of the children becoming smokers themselves [8]. This and the fact that the prevalence of smoking among Swedish 16-year-olds has been unchanged the last decade (boys 30%, girls 36%), indicates that the efforts aiming to diminish tobacco use must continue [9]. Occasionally smokers are included in these yearly investigations. Protecting children from ETS exposure is especially crucial during the first years of a child's life, since it is known to be a sensitive period in terms of the child's future health [13,14]. This is also a period of life when they spend most of their time with their parents, therefore it is important to increase the knowledge of smoking behaviour in the home and how it is influenced by different factors. Public opinion on ETS exposure has changed for the better in society and most parents are aware of the importance to protect the children from ETS. However, the major source of exposure for children is the home. Jarvis [10] recently reported that ETS exposure is not following the current positive trend in society, indicating that smoking parents are not successful in their efforts to protect their children against ETS exposure.

The physical or mental health of a parent who smokes could moderate whether the parent takes precautions to eliminate ETS from the child's environment, such as always smoking outdoors with the door closed. In research on smoking, generic instruments of health status, like SF-36 (Short Form-36) [15] are shown to be valuable to elucidate the associations between tobacco use and physical, as well as mental and social, aspects of health. This is important in smoking cessation interventions. SF-36 has been used to compare smokers and ex-smokers with never-smokers in other studies. Wilson et al [16] showed a clear trend toward lower scores, in all dimensions of quality of life, among smokers than for never-smokers and ex-smokers, and lower scores among heavy smokers than for moderate and light smokers. Considering that smoking in society probably will not be eradicated in the foreseeable future, it is crucial to study characteristics of smoking parents to find effective ways of supporting them in their efforts to minimise their children's exposure to ETS.

A better understanding of the smoking behaviours and health status of parents with different age children, compared to smoking behaviours of age-matched adults without children, will provide valuable information for planning additional intervention to reduce childhood exposure to ETS. Using a general household survey on a random sample, with focus on adults at the age when they are settling down and having children, the principal aim was to study whether having children affects adult smoking prevalence and/or smoking behaviours in the home, and how much importance survey subjects placed on protecting the indoor environment from ETS.

## Materials and methods

### Subjects

**Figure 1:**
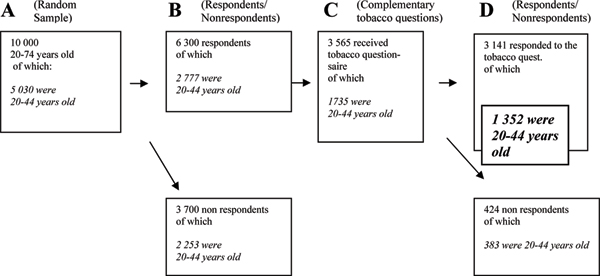
**How the final study sample (in bold text) was obtained**.

Data from a cross-sectional randomised survey, conducted in the county of Östergötland, Sweden, in 1999 were used (Figure 1). The area has 412,000 inhabitants and is a mixed area, with light and heavy industries and two towns with universities [17]. The random sample comprised 10,000 adults, 20–74 years old, obtained from the National Registration of Sweden in 1999 [17]. The sampling was done taking randomly chosen day numbers including all persons born on that date. Then every 50^th ^person was excluded until 10,000 persons remained, 5,049 men and 4,951 women; these were sent a questionnaire. After two reminders, the participation rate was 65%, with 63% usable for analysis. The sample was analysed with respect to age, sex, having dependent children, immigrant background, marital status, unemployment, education and smoking habits. The result is to be considered in accordance with a randomised group, with the exception of young men and the variable marital status (Table 1).

**Table 1:** Random sample compared to respondents, tobacco questionnaire recipients and respondents

	**Random sample**		**Complementary study**	
	10 000 randomised 20–74 years old individuals (A)	6300 responded (63%) (B)	3565 received tobacco questionnaire (C)	3141 responded to tobacco questionnaire (88%) (D)

20 – 44 years old	5030	2777	1735	1352 (study population)
				
Sex				
(% women)	48%	55%	58%	60%
				
Age groups				
20–24 years	10%	8%	9%	6%
25–34 years	20%	18%	20%	19%
35–44 years	20%	18%	20%	18%
				
Having pre-school children (<7 years)	Unknown	33%	31%	31%
				
Immigrants	10%^*a*^	10%	8%	8%
				
Marital status	Unknown			
(single)		25%	25%	24%
				
Unemployed	6%^*a*^	11%	8%	8%
				
Smokers (occ.smokers included)	19–20% (according to national reports)	19% (32%)	19% (32%)	19% (31%)
				
Education:				
Compulsury school				
(9 years)	16%^*a*^	11%	10%	10%
11 years in school	30%^*a*^	28%	29%	28%
12 years in school	24%^*a*^	36%	34%	34%
University	31%^*a*^	25%	27%	28%

^a^data from general population 20–44 years old in Östergötland

### Sample representation

Eighty-eight percent (n = 1,735) answered the complementary questionnaire about smoking behaviour. Among 20- to 44-year olds, 78% (n = 1,352) responded. A comparison was made between the random sample (A), the respondents in the main study (B), the study sample who received tobacco questions (C) and the final respondents (D) (Table 1). Women were over-represented, and the youngest age-group, 20–24 years, was under-represented, in the final study sample. Other variables were equally distributed in the different samples.

The first questionnaire consisted of seven domains dealing with demographic issues (n = 7), perceived health (n = 42), lifestyle (n = 34) and both physical and mental environment at home (n = 27) and at work (n = 44). Health status was measured with a validated instrument, the 36-item short form (SF-36) [15], encompassing 8 dimensions and 2 summary scales of physical and mental health. In 2000 a questionnaire with complementary questions about smoking behaviour was sent to 3,965 persons who, in the first questionnaire, had agreed to answer further questions (63% of the respondents). The questionnaire of the complementary study consisted of 11 items with special references to smoking behaviour, where in the home smoking was taking place, attitudes to keep the environment smoke-free, and which precautions taken by the smoker to protect the environment were considered to be effective. Answers from 20- to 44-year-old men and women were considered in this study (n = 1,735) (Figure 1). This group constituted the study sample. To strengthen the sample representation it was compared to the random sample (Table 1).

The study was approved by the local research ethics committee and performed according to the ethics code of Helsinki.

### Statistics

Data was analysed using the SPSS^® ^Version 10.1 for Windows (SPSS Inc. Chicago IL, USA). Since the data was not distributed normally, non-parametric methods were used for statistical analyses. The significance of differences in qualitative variables was tested by chi-square analyses and the Mann-Whitney U test to compare groups. Multivariate analyses were performed in order to clarify the association by each variable for being a smoker and for being an indoor smoker. Logistic regression models were created, with smoking, indoor smoking, or how important it was considered to keep the indoor environment smoke-free, as dependent variables. Education, immigration, gender, marital status, age of the children, and general health and mental health from SF-36 were independent variables.

A p-value < 0.05 was regarded as significant.

### Definitions

Smoker: Daily and occasional smokers are included.

Smoking behaviour: Active choice of places when smoking, e.g. outdoors, near an open door or the kitchen extractor fan or anywhere in the house.

Indoor smoking: smoking anywhere in the house, including standing near an open door

Outdoor smoking: smoking outdoors with the door closed.

Pre-school children: Individuals 0–6 years old. School children: Individuals 7–19 years old. Dependent children: Individuals 0–19 years old.

Immigrant: A person not born in Sweden.

## Results

### Smoking prevalence

Smoking prevalence was 31% if occasional smokers (14%) were included. Parents with preschool children (27%) smoked significantly less than parents with school children (36%) (p = 0.01), but to the same extent (p = 0.47) as adults with no dependent children (30%) (Table 2).

**Table 2:** Smoking prevalence, smoking behaviour and importance of protecting the indoor environment, related to parenthood. Percent, arithmetic mean, numbers and p-values

Numbers*	Parents with pre school-children**		Parents with only school-children**		Adults without children**		
	**(n = 387)**		**(n = 284)**		**(n = 572)**		
	Non-smoking	Smoking	Non-smoking	Smoking	Non-smoking	Smoking	p-value
Smoking prevalence		27% (106)		36% (103)			p = 0.01
		27%				30% (170)	p = 0.47
				36%		30%	p = 0.05
							
Indoor smoking (% of smokers)		28% (33)				50% (94)	p < 0.001
		28%		36% (46)			p = 0.17
							
*Missing values n = 107				36%		50% (94)	p = 0.01
							
How precautions were used among the smokers Numbers		(n = 120)		(n = 127)		(n = 190)	
							
Smoking anywhere in the home		1% (1)		3% (4)			p = 0.37
				3%		5% (10)	p = 0.58 (Fisher)
		1%				5%	p = 0.06 (Fisher)
							
Smoking near the kitchen fan		12% (14)		17% (22)			p = 0.28 (Fisher)
				17%		12% (23)	p = 0.14 (Fisher)
		12%				12%	p = 1.0 (Fisher)
							
Smoking near an open door		9% (11)		10% (13)			p = 0.28 (Fisher)
				10%		20% (38)	p = 0.03
		9%				20%	p = 0.02
							
Importance of protecting the indoor environment (range 1–4; 4 most important)							
							
Mean values	3.98	3.83	3.97	3.74	3.93	3.68	p < 0.001 between non-smokers and smokers in all three groups

**a family was categorised according to the age of their youngest child

In logistic regression models, education, gender and self-reported mental health (in SF-36) were the only variables with significant association to smoking (Table 3).

**Table 3:** Logistic regression models showing variables with association to smoking, indoor smoking, and how important it is considered to keep indoor environment smoke-free. Odds ratios (OR), 95% Confidence interval (CI), and p-values

Independent variables	Dependent variables Smoking			Indoor smoking			Important to protect indoor environment		
	OR	95% CI	p-value	OR	95% CI	p-value	OR	95% CI	p-value
Education	0.72	0.63–0.83	<0.001	1.18	0.94–1.48	0.16	1.53	1.25–1.88	<0.001
Gender	1.40	1.06–1.79	0.02	0.94	0.60–1.46	0.78	1.19	0.81–1.73	0.38
Marital status	1.00	0.78–1.27	0.97	1.16	0.81–1.66	0.43	1.09	0.78–1.52	0.62
									
Age of the children*									
Pre school	Ref			Ref			Ref.		
School	1.35	0.96–1.90	0.08	1.36	1.24–3.44	0.01	0.56	0.31–0.98	0.04
No children	1.15	0.84–1.56	0.38	2.95	1.74–4.98	<0.001	0.37	0.22–0.61	<0.001
Immigrant status	0.81	0.52–1.27	0.36	2.87	1.47–5.62	0.002	1.38	0.72–2.68	0.33
General health (SF-36)	1.00	0.99–1.01	0.69	1.01	1.00–1.02	0.08	1.01	1.00–1.02	0.34
Mental health (SF-36)	0.99	0.98–0.99	<0.01	1.01	1.00–1.02	0.09	1.00	1.00–1.01	0.75

*a family was categorised according to the age of their youngest child

### Smoking behaviour

Sixty percent of all smokers smoked outdoors. Parents with preschool children (72%) and school children (64%) smoked outdoors significantly more than adults without children (50%) (Table 2). Smoking near an open door was more common among adults without children, other precautions were used to about the same extent in the three groups (Table 2).

Logistic regression models showed that indoor smoking was associated with being an immigrant, and having dependent children was positively associated with outdoor smoking (Table 3).

### Attitudes to protect the home from tobacco smoke

All respondents considered it important to keep the indoor environment smoke-free. Non-smokers found it more important than smokers, and smoking parents with preschool children found it significantly more important than did adults without children (Table 2).

Logistic regression models showed that the age of the children and education were variables with significant impact on how important it was considered to keep the environment smoke-free (Table 3). When asked which precautions were effective, 53% named smoking outdoors with the door closed, 10% added changing clothes, whereas 3% thought smoking near the kitchen fan or near an open door, or airing the room, respectively, were sufficient. Thirty-three percent declared that it is impossible to protect the indoor environment from tobacco smoke as long as someone is smoking in the household.

### Differences in life style and health-related quality of life between smoking and non-smoking parents

In the study group, 58% had dependent children. No differences in alcohol consumption were found between smoking and non-smoking parents, but non-smokers reported significantly more physical activity.

The instrument, SF-36 [15], measuring self-perceived health, revealed differences between smokers and non-smokers. Factors measuring physical health such as "role-physical," "physical functioning," and "bodily pain" did not differ, but social and mental factors such as "general health," "vitality," "social functioning," "role-emotional," and "mental health" all showed significantly higher scores among non-smokers than for smokers (Table 4). Logistic regression models with the 8 domains from SF-36 as independent variables showed that mental health was significantly associated (OR 0.99 CI 0.97–1.00, p = 0.03) with smoking prevalence as well as smoking behaviour (OR 1.03, CI 1.01–1.05, p = 0.002). None of the domains in SF-36 were of importance for how important it was considered to protect the indoor environment

**Table 4:** Score differences in health-related quality of life, in the 8 domains of SF-36, between smoking and non-smoking, 20–44 years old, adults with and without children. Mean scores (range), p-values

		Physical functioning	Role-physical	Bodily pain	Genera health	Vitality	Social functioning	Role-emotional	Mental health
All n = 1352	Smokers (n = range 374–379)	93 (5–100)	84 (0–100)	76 (0–100)	74 (5–100)	61 (0–100)	84 (0–100)	80 (0–100)	74 (0–100)
	Non-smokers (n = range 848–862)	95 (0–100)	87 (0–100)	79 (0–100)	79 (0–100)	66 (0–100)	89 (0–100)	87 (0–100)	80 (12–100)
	p-value	<0.01	0.03	0.04	<0.01	<0.01	<0.01	<0.01	<0.01
									
All parents having dependent children n = 750	Smokers (n = range 205–209)	93 (5–100)	84 (0–100)	74 (0–100)	75 (15–100)	59 (0–100)	84 (0–100)	83 (0–100)	75 (0–100)
	Non-smokers (n = range 453–461)	94 (0–100)	87 (0–100)	77 (0–100)	80 (0–100)	66 (0–100)	89 (12.5–100)	89 (0–100)	82 (20–100)
	p-value	0.12	0.09	0.07	<0.01	<0.01	<0.01	<0.01	<0.01
									
All parents having pre-school Children n = 422	Smokers (n = range 104–106)	95 (45–100)	86 (0–100)	77 (0–100)	77 (20–100)	61 (0–100)	85 (0–100)	82 (0–100)	76 (16–100)
	Non-smokers (n = range 276–280)	94 (0–100)	90 (0–100)	79 (12–100)	80 (10–100)	66 (0–100)	90 (12.5–100)	90 (0–100)	82 (20–100)
	p-value	0.24	0.06	0.30	0.04	0.08	0.02	<0.01	<0.01
									
All parents having school children (n = 328)	Smokers (n = range 102–103)	91 (5–100)	82 (0–100)	70 (12–100)	73 (15–100)	57 (0–100)	83 (0–100)	83 (0–100)	73 (0–100)
	Non-smokers (n = range 179–181)	93 (0–100)	82 (0–100)	74 (0–100)	79 (0–100)	67 (0–100)	88 (12.5–100)	87 (0–100)	81 (20–100)
	p-value	0.42	0.84	0.24	0.03	<0.01	0.08	0.18	<0.01
									
Adults without children n = 602	Smokers (n = range 168–170)	94 (10–100)	84 (0–100)	78 (0–100)	74 (0–100)	63 (0–100)	84 (0–100)	77 (0–100)	74 (0–100)
	Non-smokers (n = range 393–400)	96 (0–100)	87 (0–100)	81 (0–100)	78 (15–100)	67 (0–100)	89 (0–100)	84 (0–100)	79 (12–100)
	p-value	0.01	0.25	0.37	0.05	0.04	0.02	<0.01	0.04

In order to elucidate whether the age of the children or having no children made any differences, analyses were done for the three groups separately (Table 4). The results indicate a power effect, with smaller groups showing fewer statistically significant differences. However, "mental health" from SF-36 remained different in all groups, with lower scores for smokers. This was also the case for "general health" in all groups apart from the group without children, the group with the lowest mean age, 29 years. Parents of preschool children and parents of only school children had a mean age of 33 and 39 years respectively.

## Discussion

Our results indicate that parenthood had an impact on attitudes toward protecting the environment from ETS and smoking behaviour, but having dependent children did not show a significant impact on smoking prevalence. Smoking parents showed lower scores in several domains in self-perceived health-related quality of life measured with SF-36.

The participant rate was 63% in the main study; today it is difficult to get higher response rates in epidemiological studies like this, in Scandinavia [18,19]. The analyses showed good agreement between the random sample (A), the respondents in that sample (B), the sample for the complementary study (C), and the respondents of this (D). The prevalence of daily smokers in the geographical area of the study was also equal to the current Swedish national prevalence. Education, known to influence smoking prevalence [1], was about the same in the study population as in the general population. Smoking prevalence and prevalence of having preschool children were also equivalent between the groups.

The age groups for parents were chosen because they are the ages when starting a family and having children is most common. Older and very young parents were not included, and judging from other studies [20] this may have resulted in an underestimation of smoking prevalence among parents. These groups are, however, small in Sweden [21] and thus did not impair the study results to any great extent. Other limitations are that women during their first pregnancy have been misclassified as non-parents in the analyses, and during pregnancy and infancy smoking habits often are unstable.

The instruments used in this study were not tested for validity or reliability. However, the main study comprised widely used questions as well as a validated instrument (SF-36), and the complementary questions for this study were designed, scrutinised by specialists, and pre-tested. We also had to rely on statements from those who are responsible for the ETS exposure, and since the issue must be considered delicate, this could have underestimated smoking prevalence and overestimated the use of precautions [11].

The smoking prevalence among parents of pre-school children in this study was compared to annual statistics from The National Swedish Board of Health and Welfare [22]. In 1998, national statistics on smoking among mothers and fathers [3] showed that 12% of mothers and 15% of fathers of 8-month-old children were registered as smokers in the children's records at the Child Health Care units. The number of smokers in this report might be underestimated since missing data tends to exceed the number of reported smokers. The higher smoking prevalence in our study, 27% among parents of preschool children, can also be explained by the inclusion of occasional smokers and parents of older children, as well as by the fact that the questions were asked anonymously and the respondents were not asked in their capacity as parents. This might have had an impact on the result since the combination of smoking and parenthood has become socially unacceptable [11].

The higher smoking prevalence among parents of school children might be associated with the less intensive tobacco prevention work in antenatal clinics as well as in Child Health Care at the time when this group of parents attended these, and thus might be considered as a positive evaluation of this work.

Having children was significantly associated with outdoor smoking. The younger the children, the more important outdoor smoking seemed to be, which is in accordance to Eriksen and Bruusgaard's study in Norway [23]. This may be a result of the increased awareness and efforts among nurses in Child Health Care, as well as low tolerance of passive smoking in general.

Non-smokers, parents of preschool children and higher-educated adults with and without children considered it more important to protect the indoor environment from ETS. However, 33% of the study population stated that it was impossible to protect the home from ETS as long as a single member of the household is a smoker, regardless of the precautions taken. This kind of attitude has been shown to lessen the motivation to take protective measures and encourages a more fatalistic mindset [24]. The results of this study can hardly support or refute this theory, but stress the need for further research on the effectiveness of different precautions.

The use of SF-36 gave valuable information behind the statistics on a number of smokers and non-smokers. Differences in health-related quality of life between smoking and non-smoking parents of dependent children were obvious with SF-36. In this study we have compared young (20–44 years) parents of dependent children and found that smokers had significantly lower scores in "general health," "vitality," "social functioning," "role-emotional." and "mental health" than non-smokers, results indicating that smoking can not be seen as an isolated issue. In many cases it is one problem among others, but one important question, raised by these results, is the impact of quality-of-life on the ability to adapt a protecting smoking behaviour in the home. However, logistic regression models did not show any significant impact of the 8 SF-36 domains on how important it was considered to protect indoor environment. Only "mental health" had an impact on smoking prevalence and smoking behaviour, indicating that the ambitions are the same in all groups, but the ability to fulfil one's intentions is less when variables measured in the domain "mental health" are lower scored. Other studies have shown that in a general population smoking prevalence is higher among groups with reduced mental health [25]. Therefore it is necessary to consider the special needs, social, political and economic, of these groups when planning strategies to support these parents in their efforts to protect their children from tobacco smoke.

This study shows that adults, with or without dependent children, smoke to the same extent. The results indicate that national surveys of smoking prevalence give a good approximation of the percentage of children with parents who smoke. Preschool children spend most of their time in the home. In this study, smoking occurred in 27% of the households with preschool children, thus putting these children at risk of ETS exposure. However, the knowledge of the hazardous health effects of ETS on both smokers and non-smokers seems to have made most parents aware of the importance of protecting the children from ETS [23,26,27].

This study indicates that parenthood is associated with smoking behaviour and attitudes toward passive smoking, but not to smoking prevalence. It points out the need for further research on how effective commonly used precautions are, as well as finding new ways of protecting the children from ETS. It is also important to find out how interventions, aiming to support smoking parents in their efforts to protect their children from ETS, should be designed.

## Competing interests

The authors declare that they have no competing interests.
